# Supplementary material for: FGF Modulates the Axial Identity of Trunk hPSC-Derived Neural Crest but Not the Cranial-Trunk Decision
Source: Stem Cell Reports. 2019 May 14;12(5):920–33. doi: 10.1016/j.stemcr.2019.04.015 (PMC6524753; doi:10.1016/j.stemcr.2019.04.015)
Supplement: Document S1. Figures S1–S3 and Tables S1 and S2 [file mmc1.pdf]

Stem Cell Reports, Volume *12*

Supplemental Information

FGF Modulates the Axial Identity of Trunk hPSC-Derived Neural Crest but Not the Cranial-Trunk Decision

James O.S. Hackland, Patrick B. Shelar, Nabjot Sandhu, Maneeshi S. Prasad, Rebekah M. Charney, Gustavo A. Gomez, Thomas J.R. Frith, and Martín I. García-Castro

Table S1. List of primary antibodies

Target	Source/Cat#	Dilution
SOX10	Santa Cruz/271163	1:200
PAX7	DSHB	1:20
Peripherin	Sigma-Millipore/ab1530	1:200
Peripherin	Santa Cruz/377093	1:200
ISL1	DSHB/39-4D5	1:100
Β-Tubulin	Santa Cruz/365791	1:300
BRN3a	Santa Cruz/8429	1:100
ASCL1	Santa Cruz/374104	1:1000
PHOX2B	Santa Cruz/376997	1:500
Tyrosine Hydroxylase (TH)	Santa Cruz/25269	1:500
S100B	Santa Cruz/393919	1:50
Vimentin	Sigma-Millipore/MAB3400	1:100
SMA	Sigma/A2547	1:100
HOXC9	Santa Cruz/81100	1:500
Bra(T)	R&D/AF2085	1:200
CDX2	DSHB/PCRP-1A3	1:250
MITF	R&D/AF5769	1:50

Table S2. List of primers

Transcript	Forward	Reverse
GAPDH	ATGGGTGTGAACCATGAGAA	GTTGTCATGGATGACCTTGG
SOX10	GAGGCTGCTGAACGAAAGTGA	GCGGCCTTCCCGTTCT
TFAP2A	GAGAGTAGCTCCACTTGGGTG	GTCGTGACGGTCCTCGC
SNAI2	CAGACCCTGGTTGCTTCAAG	GAGCCCTCAGATTTGACCTG
FOXD3	TCATCACCATGGCCATCCT	GGAAGCGGTTGCTGATGAAC
PAX3	GAACCCGGGCATGTTCAG	ACGGCACGGTGTTTCGA
PAX7	GCGACTCCGGATGTAGAGAA	ATCCTTCAGCAGCCTGTCC
PAX6	ACCCGGCAGAAGATTGTAGA	ACTCACATCCGTTGGACA
OCT4	TGCAGCTTAGCTTCAAGAACATG	TCAGCTTCCTCCACCCACTT
BRA(T)	AGGCTCCCGTCTCCTTCAGCAA	TGGCTGGTGATCATGCGCTGT
SOX2	TGCGAGCGCTGCACAT	GCAGCGTGTACTTATCCTTCTTCA
CDX2	GGAACCTGTGCGAGTGGAT	TGAAACTCCTTCTCCAGCTCC
NKX1.2	AAAGTTTGGCGGAGGTCGAA	GCAGCATCAGGGGTCTCC
OTX2	GAGAGGAGGTGGCACTGAAAA	GTTGTTGGCGGCACTTAGC
HOXB4	TACCCCTGGATGCGCAAAGTTC	TGGTGTTGGGCAACTTGTGG

HOXC5	ACAGATTTACCCGTGGATGAC	AGTGAGGTAGCGGTTAAAGTG
HOXC6	ATGAATTCGCACAGTGGGGT	TCCTTCTCCAGTTCCAGGGT
HOXC8	GATGAGACCCCACGCTCCG	TTCTAGTTCCAAGGTCTGATACCG
HOXC9	AGCACAAAGAGGAGAAGGC	CGTCTGGTACTTGGTGTAGG
HOXA10	GGATTCCCTGGGCAATTCCAAA	CAGTGTCTGGTGCTTCGTGT
HOXA11	GGCGGCTCCAGTGGC	CGCTGAAGAAGAACTCCCGT
HOXD13	ACGCTGGCTAACGGGTG	TTAGAGCCACATCCCCTGGA

Figure S1

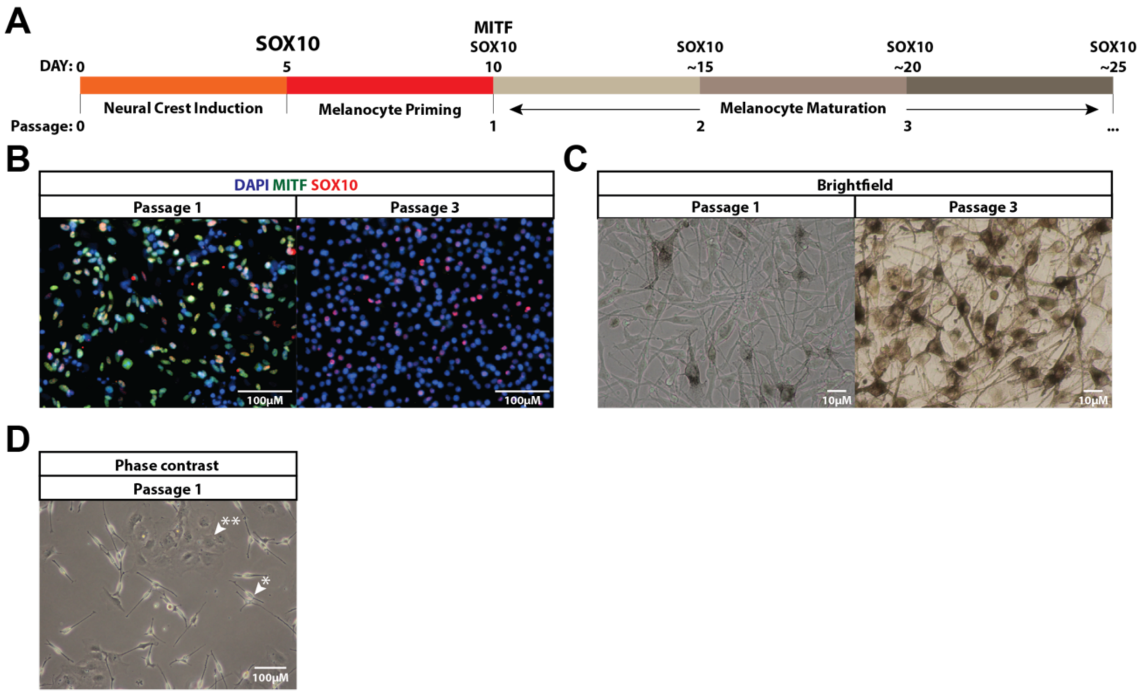

Figure S1. Melanocyte induction.

Related to Figure 2. (A) Schematic demonstrating melanocyte induction protocol. After 5 days of neural crest induction culture conditions were exchanged for a priming medium containing BMP4 and EDN3. After a further 5 days cells were detached and seeded into melanocyte maturation conditions (see Methods section). Subsequent passaging was carried out when cultures became highly confluent, approximately every 5 days. (B, C) After primed cells were detached and seeded into maturation conditions expression of MITF and SOX10 could be observed and a minority of cells exhibited pigmentation. Continued culture in these conditions resulted in a loss of MITF expression and a dramatic increase in the proportion of pigmented cells. Images of pigmented cells (C) were taken using a 20x objective on a Nikon TS100 inverted microscope with the condenser set to brightfield (i.e. no phase ring). (D) Low magnification phase contrast imaging (i.e. using a phase ring) at passage 1 reveals glowing, motile melanocyte progenitors (*) and low flat cells not of the melanocyte lineage (**). The latter terminally differentiate and are eventually outgrown and lost with passaging. Images shown are from melanocyte induction using Miff-1-derived neural crest (B, C) and H1-derived neural crest (D).

Figure S2

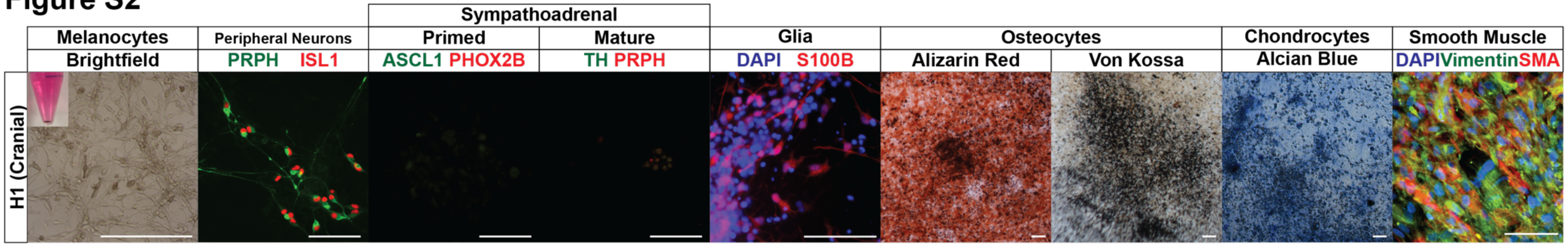

Figure S2. Terminal differentiation of cranial neural crest

Related to Figure 2. Terminal differentiation of cranial neural crest generated using a previously published protocol (Leung et al., 2016).

Figure S3

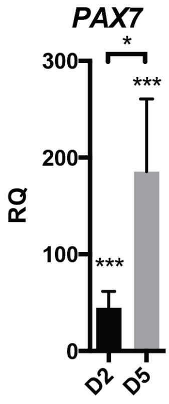

Figure S3. *PAX7* expression in day 2 neural crest progenitors

Related to Figure 3. qPCR data depicting expression of *PAX7* at day 2 and day 5 of neural crest induction using the W2B3 protocol in H1.
