# Supplementary material for: FGF Modulates the Axial Identity of Trunk hPSC-Derived Neural Crest but Not the Cranial-Trunk Decision
Source: Stem Cell Reports. 2019 May 14;12(5):920–33. doi: 10.1016/j.stemcr.2019.04.015 (PMC6524753; doi:10.1016/j.stemcr.2019.04.015)
Supplement: Document S2. Article plus Supplemental Information [file mmc2.pdf]

Stem Cell Reports
Article
OPEN ACCESS

FGF Modulates the Axial Identity of Trunk hPSC-Derived Neural Crest but Not the Cranial-Trunk Decision

James O.S. Hackland,[1,*] Patrick B. Shelar,[1] Nabjot Sandhu,[1] Maneeshi S. Prasad,[1] Rebekah M. Charney,[1] Gustavo A. Gomez,[1] Thomas J.R. Frith,[2] and Martín I. García-Castro[1,*]
[1]University of California Riverside, Department of Biomedical Sciences, Riverside, CA 92521, USA
[2]University of Sheffield, Department of Biomedical Science, Sheffield S10 2TN, UK
*Correspondence: hacklandj@gmail.com (J.O.S.H.), martin.garcia-castro@ucr.edu (M.I.G.-C.)
https://doi.org/10.1016/j.stemcr.2019.04.015

SUMMARY

The neural crest is a transient embryonic tissue that gives rise to a multitude of derivatives in an axially restricted manner. An *in vitro* counterpart to neural crest can be derived from human pluripotent stem cells (hPSCs) and can be used to study neural crest ontogeny and neurocristopathies, and to generate cells for therapeutic purposes. In order to successfully do this, it is critical to define the specific conditions required to generate neural crest of different axial identities, as regional restriction in differentiation potential is partly cell intrinsic. WNT and FGF signaling have been implicated as inducers of posterior fate, but the exact role that these signals play in trunk neural crest formation remains unclear. Here, we present a fully defined, xeno-free system for generating trunk neural crest from hPSCs and show that FGF signaling directs cells toward different axial identities within the trunk compartment while WNT signaling is the primary determinant of trunk versus cranial identity.

INTRODUCTION

Human pluripotent stem cells (hPSCs) can be differentiated *in vitro* into a wide variety of specific cell types. Research into this process has broadened our understanding of disease (Miller et al., 2013; Okuno et al., 2017), has been used to model developmental processes (Funa et al., 2015), and has made the derivation of cells for therapeutic use a reality (Da Cruz et al., 2018). One cell type that can be generated in this manner is the *in vitro* counterpart to embryonic neural crest. After formation at the border of the neural plate, neural crest cells migrate throughout the developing embryo, differentiating into a wide variety of terminal derivatives (His, 1868; Le Lievre and Le Douarin, 1975; Marshall, 1878; Simões-Costa and Bronner, 2015). Any disruption to this process can lead to a myriad of diseases, disorders, and syndromes with divergent characteristics that are collectively known as neurocristopathies (Bolande, 1974). For this reason, the study of hPSC differentiation into neural crest has received a great deal of attention. The methods by which hPSCs can be differentiated into neural crest have evolved considerably from co-culture-based strategies (Pomp et al., 2005) and protocols containing animal-derived components (Lee et al., 2010) or mechanical purification steps (Menendez et al., 2011) to highly defined approaches that can generate cells in a short period of time (Hackland et al., 2017; Leung et al., 2016; Tchieu et al., 2017).

In the embryo, the differentiation potential of neural crest is regionally restricted along the rostral-caudal axis (Chibon, 1967; Le Douarin et al., 2004; Lwigale et al., 2004; Nakamura and Ayer-le Lievre, 1982; Rothstein et al., 2018). The exact nature of this restriction is still poorly understood, and it is possible that cells from some regions may retain the ability to form otherwise restricted derivatives if they are subjected to the correct signals (Le Douarin et al., 2004; Le Douarin and Teillet, 1974; McGonnell and Graham, 2002). *HOX* genes are key molecular regulators of this process, and the *HOX* code of a cell is a good indicator of axial identity (Deschamps and Duboule, 2017; Parker et al., 2018). *In vitro*, the lack of *HOX* gene expression (or presence of anterior *HOX* expression), coupled with an ability to differentiate into osteocytes and chondrocytes, suggests that hPSC-derived neural crest have a cranial identity by default (Hackland et al., 2017; Lee et al., 2010; Leung et al., 2016; Mica et al., 2013). Treatment with retinoic acid can be used to induce the expression of vagal *HOX* genes, but expression of posterior, trunk *HOX* genes cannot be induced in this manner (Fattahi et al., 2016; Frith et al., 2018; Mica et al., 2013).

In vivo, caudal tissues are derived from a bipotent axial progenitor cell type that exists within the node-streak border (NSB) and caudal lateral epiblast (CLE). This axial progenitor expresses both *SOX2* and *Brachyury (T)*, has the potential to form either neural or mesodermal derivatives (Cambray and Wilson, 2002; Gont et al., 1993; Tzouanacou et al., 2009; Wymeersch et al., 2016), and can be generated *in vitro* from both mouse and human embryonic stem cells (hESCs) (Gouti et al., 2014; Lippmann et al., 2015; Tsakiridis et al., 2014; Tsakiridis and Wilson, 2015; Turner et al., 2014). There is evidence to suggest that neural crest cells can also be derived via this route (Catala et al., 1995; Denham et al., 2015; Frith et al., 2018; Tzouanacou et al., 2009; Wymeersch et al., 2016), although this is not the only potential source of neural crest in the trunk. A

 © 2019 The Authors.
This is an open access article under the CC BY license (http://creativecommons.org/licenses/by/4.0/).

groundbreaking publication by Denham et al. (2015) described a protocol by which neural crest aggregates can be generated that lack expression of anterior markers and pass through a SOX2/Brachyury positive intermediate state. Although these cells remain capable of differentiating into osteocytes and chondrocytes, and expression of posterior *HOX* genes was not presented, it is likely that the cells generated in this way exhibit a trunk neural crest identity. There have been further publications describing trunk neural crest induction using similar cell aggregation techniques (Abu-Bonsrah et al., 2018; Kirino et al., 2018), and recently it has been shown by Frith et al. (2018) that axial progenitors derived from hPSCs in a WNT and FGF-dependent manner using a previously published system (Gouti et al., 2014) can be directed toward a neural crest fate. Despite this, the exact roles played by WNT and FGF signaling in inducing a posterior identity in the neural crest are still poorly understood, and there remains a need to generate trunk neural crest in a way that does not involve passaging or aggregation steps and in a medium that is free from BSA.

Here, we describe a new system, termed W2B3, for the differentiation of hPSCs into an *in vitro* counterpart to trunk embryonic neural crest. The system is fully defined, xeno-free, and robust across five different hPSC lines. In addition to this, we identify distinct roles for both WNT and FGF signaling during the first 2 days of differentiation: WNT directs cells toward a cranial or trunk identity in a binary, dose-dependent manner, and FGF modulates HOX gene expression within the trunk compartment. This allows for easy generation of cells corresponding to a wide range of axial subtypes, including anterior (cervical) or posterior (thoracic) trunk neural crest via manipulation of the FGF signaling environment during this period. High levels of FGF can even induce the expression of sacral *HOX* genes, although *SOX10* expression is lost. Axial identity is demonstrated by distinct gene expression profiles as well as functional analysis in comparison with an established cranial neural crest induction protocol (Gomez et al., 2019a; Leung et al., 2016). In agreement with the studies highlighted above, we also report the existence of an intermediate cell type that expresses *Brachyury* and *SOX2* and as such resembles the axial stem cell population that resides in the NSB and CLE of the developing embryo.

RESULTS

To develop a robust neural crest induction system that is effective across multiple different hPSC lines, we set out to combine elements of other approaches that allow for speed and efficiency, but achieve it in a fully defined and xeno-free environment. To this end, we combined early restricted WNT activation and sustained inhibition of the ROCK pathway (Gomez et al., 2019a; Leung et al., 2016) with tightly controlled BMP activation using top-down inhibition (TDi) in a fully defined environment (Hackland et al., 2017). During this process, it was determined that BMP control using TDi was only required for the second portion of neural crest induction (days 2–5; Figure 1A) and that TGF-β inhibition was not required as reported in Leung et al. (2016) (data not shown). Thus, this new hybrid approach utilizes WNT stimulation for the first 2 days and controlled BMP stimulation for the following 3 days, leading us to refer to it as the "W2B3" protocol. When we applied this approach using a fully defined, xeno-free medium based on DMEM F12 supplemented with N2, we were able to achieve highly efficient (80%–90% SOX10$^+$) neural crest induction across five different hESC and hiPSC lines (Figure 1B). After 5 days of induction, all cell lines tested exhibited expression of neural crest-associated factors (*TFAP2A*, *SNAI2*, *FOXD3*, *PAX3*, *PAX7*, *SOX10*), while expression of the neural-associated transcription factor *PAX6* remained low (H1-ΔcT pluri 15.5; NC 13.0) and the pluripotency marker *POU5F1* was downregulated (Figure 1C).

In vivo neural crest cells have the potential to differentiate into a wide variety of different cell types. Some of these derivatives are restricted to specific axial locations; such as the mesenchymal derivatives of the cranial neural crest, or the sympatho-adrenal derivatives of the trunk neural crest (Chibon, 1967; Lwigale et al., 2004; Le Douarin et al., 2004). To functionally characterize the W2B3-derived neural crest-like cells from all five hPSC cell lines (three hESC, two hiPSC), we subjected them to a series of different conditions designed to direct them toward terminal derivatives. As with previously published protocols, W2B3-derived neural crest cells are capable of differentiating into sensory neurons that express peripherin, β-tubulin, ISL1, and BRN3a, as well as glia that express SOX10 and S100B (Figure 2). They are also able to differentiate into smooth muscle, expressing vimentin and SMA, and osteocytes that deposit calcium and can be stained with alizarin red and Von Kossa (Figure 2). However, unlike previous approaches, we were unable to generate chondrocytes. Further to this, neither of our previously published protocols reported the successful formation of terminally differentiated, pigmented melanocytes (Leung et al., 2016; Hackland et al., 2017), and the only published example of this required cell sorting for a small (10%) c-kit positive subpopulation of cells (Mica et al., 2013). Here, we show that W2B3-derived neural crest responds to a simple melanocyte induction protocol with no cell-sorting step by differentiating into pigmented melanocytes (Figure 2) via a MITF and SOX10 positive intermediate (Figure S1). Cranial neural crest generated using a previously published approach

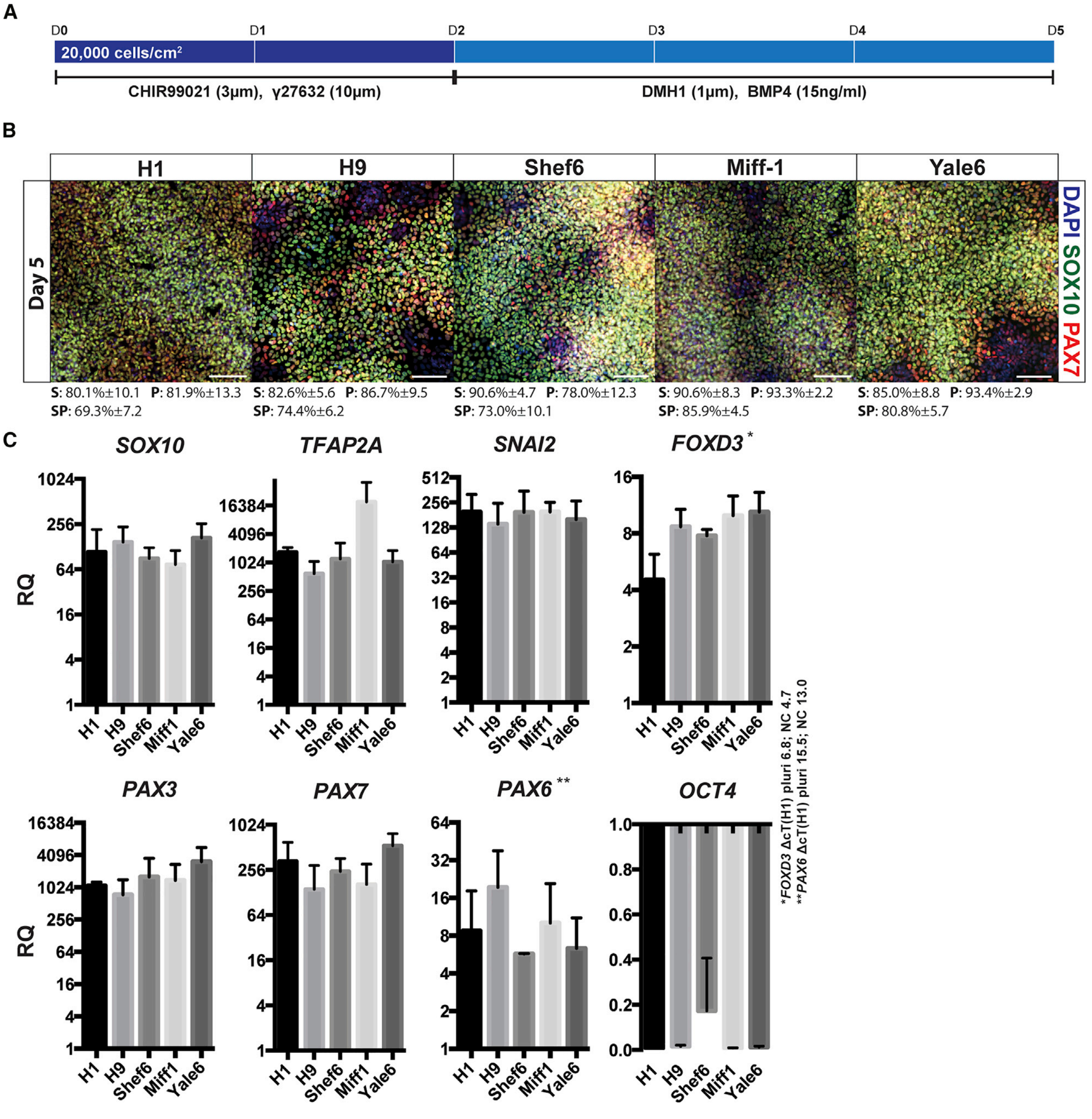

Figure 1. Robust Neural Crest Induction in Fully Defined Conditions

(A) Schematic depicting neural crest induction with the W2B3 protocol.

(B) Immunofluorescence microscopy demonstrating expression of neural crest-associated transcription factors SOX10 and PAX7 in five different hPSC lines. Quantification is calculated from three independent experiments and presented below each image. S, SOX10 positive; P, PAX7 positive; SP, SOX10/PAX7 positive. Scale bar represents 100 µm.

(C) qPCR data showing change in expression of neural crest-associated genes *SOX10*, *TFAP2A*, *SNAI2*, *FOXD3*, *PAX3*, *PAX7*, neural-associated gene *PAX6*, and stem cell marker *OCT4* (*POU5F1*). Gene expression is presented relative to initial undifferentiated hPSCs (RQ). Examples of the difference in cycle threshold with housekeeping gene (ΔcT) are presented for *FOXD3* and *PAX6* in H1, marked * and **, respectively.

(Leung et al., 2016) also responded in this manner, although extended culture in melanocyte maturation conditions was required (Figure S2). Finally, unlike our previous cranial neural crest protocol, the W2B3 system produces neural crest that can generate cells of the sympatho-adrenal lineage. Precursors expressing ASCL1

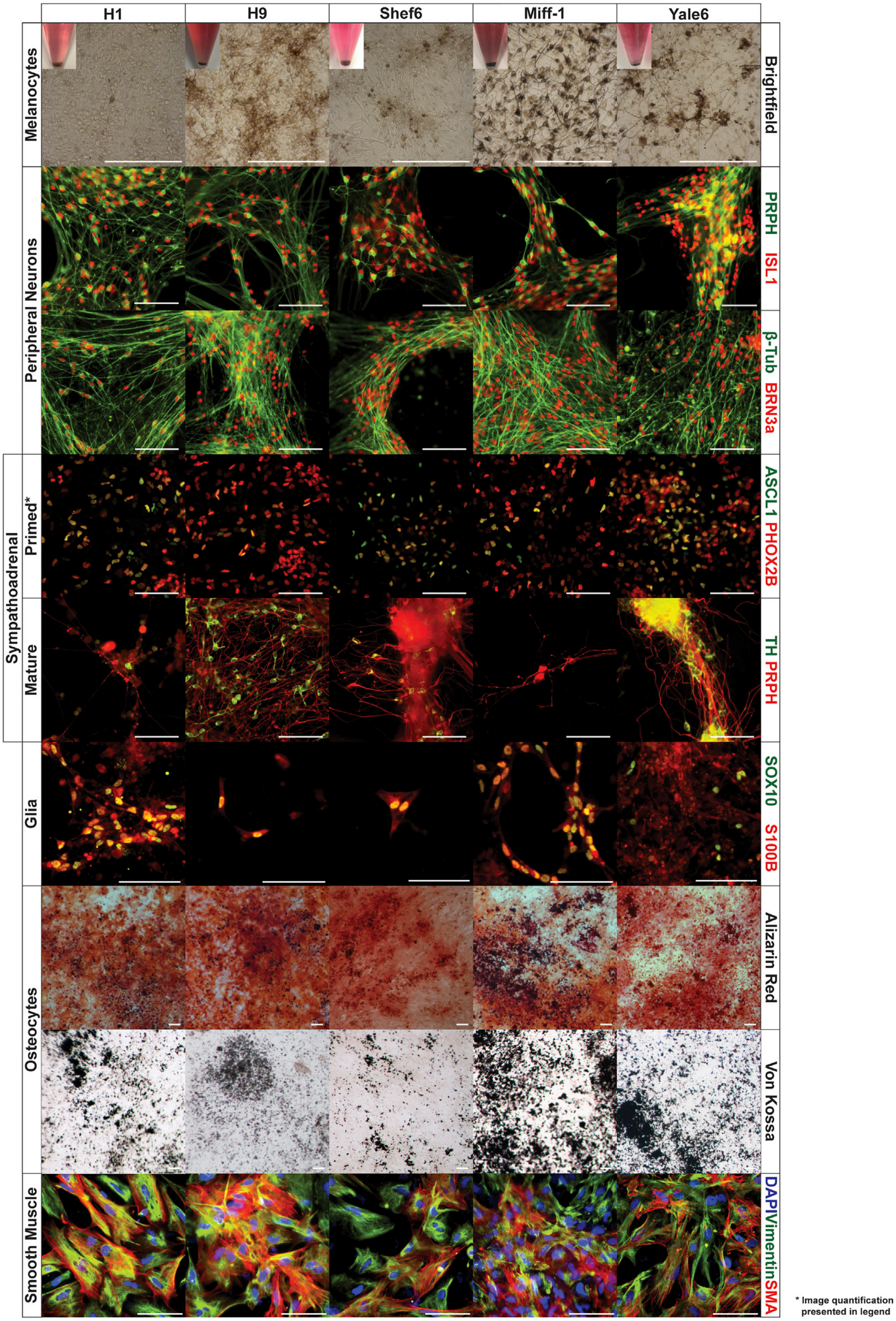

(legend on next page)

and PHOX2B were generated, as well as peripherin+/tyrosine hydroxylase+ neurons (Figure 2). Differentiation of cranial neural crest in the same conditions resulted in cells negative for ASCL1 and PHOX2B that died upon transfer to maturation conditions rather than forming neurons (Figure S2).

The distinct functional potential of the cells generated using the W2B3 system when compared with that of previously published protocols is intriguing. Specifically, the capacity to make sympatho-adrenal cells, albeit with variable efficiency, but the lack of chondrocyte formation suggests that they correspond to trunk neural crest rather than cranial neural crest as has been previously published (Hackland et al., 2017; Lee et al., 2010; Leung et al., 2016; Menendez et al., 2011; Mica et al., 2013; Tchieu et al., 2017). In support, the trunk markers *CDX2* and *HOXC9* are widely co-expressed with *SOX10* on day 5 of induction (Figures 3A and 4D), and qPCR shows that *HOXB4*, *HOXC5*, and *HOXC9* are all upregulated by day 5, although to varying degrees between replicates (Figure 3B).

There is evidence to suggest that trunk neural lineages, unlike those of the cranial region, pass through an intermediate progenitor state that exists at the border of the primitive streak and has the potential to form both neural and mesodermal derivatives (Cambray and Wilson, 2002; Gont et al., 1993; Gouti et al., 2014; Tzouanacou et al., 2009; Wymeersch et al., 2016). *In vivo* these axial progenitors are known to express the neural marker *SOX2* and the mesodermal marker *Brachyury* (*T*) in a heterogeneous fashion (Wymeersch et al., 2016). To determine if the neural crest cells generated using the W2B3 system pass through such a state, we analyzed cultures at day 2 of induction and found that most cells were positive for both SOX2 and Brachyury (Figure 3C). There was also a significant increase in *PAX7* transcripts at this stage (Figure S3), although these levels are approximately two-thirds of those found consistently at day 3 when robust PAX7 immunofluorescent signal can be detected for the first time (data not shown). In an effort to assess the capacity of the cells to differentiate into mesodermal derivatives, the controlled BMP conditions used between days 2 and 5 were replaced by sustained WNT (CHIR99021) and FGF (FGF2) exposure. In these conditions, expression of the mesodermal marker *Brachyury* was observed at day 5 (Figures 3D and 3E). In contrast to this, maintenance of WNT and FGF signaling during cranial neural crest formation did not lead to Brachyury expression after 5 days of culture (Figure 3E). However, qPCR data demonstrated that *SOX2* is also highly expressed in this condition, while other mesoderm markers *MSGN1* and *TBX6* are not (Figure 3F).

In the developing embryo, FGF and WNT signals are reported to play key roles in induction of neural crest, coordination of posterior identity, and formation of axial progenitors. To our surprise, we found that treatment of induction cultures with the FGF inhibitor PD17 (200 nM) during the first 2 days of induction did not have a significant effect on *SOX10* expression at day 5 (Figures 4B and 4D). FGF inhibition also did not result in a significant change in *SOX2* expression at day 2 of induction but did cause a decrease in Brachyury expression. Conversely, treatment during the same period with FGF2 (10 ng mL^{-1}) prevented *SOX10* induction by day 5, resulted in a dramatic downregulation of *SOX2* at day 2, and promoted *Brachyury* expression to very high levels on day 2, although the latter was not retained to day 5 (Figures 4A–4C).

Manipulation of FGF signaling during the first 48 h of induction also affected the expression of axial markers. The trunk marker *CDX2* was upregulated to levels approximately 10,000-fold higher than in pluripotent cells at day 2 of induction using the W2B3 protocol. Although expression of *CDX2* was not significantly different at day 2 in the presence of FGF2, by day 5, *CDX2* expression had been significantly retained in the FGF-treated condition compared with the untreated control (Figure 4B). In contrast to this, treatment with PD17 appeared to switch off *CDX2* faster than in the control. The axial progenitor marker *NKX1.2* was expressed early during induction but expression was lost by the time neural crest identity was acquired, as has been previously observed (Figure 4B; Frith et al., 2018). Expression of *OTX2* was not observed in either condition, in contrast to the cranial neural crest induction system published by Leung et al. (2016) (Figures 4B and 4D). Assessment of *HOX* gene expression found that those associated with the cervical and thoracic regions of the embryo were all upregulated in the W2B3 system, but sacral *HOX* genes were not expressed. However, in the FGF condition, expression of the sacral *HOX* genes *HOXA10*,

Figure 2. Terminal Differentiation of Putative Neural Crest Generated Using the W2B3 System
Evidence is presented for the differentiation of W2B3-derived neural crest from five different hPSC lines into multiple terminal derivatives, including melanocytes (pigmentation), peripheral neurons (peripherin, β-tubulin, ISL1, and BRN3a), sympatho-adrenal lineages (ASCL1, PHOX2B, tyrosine hydroxylase and peripherin), glia (S100B and SOX10), osteocytes (alizarin red and Von Kossa stains), and smooth muscle (vimentin and SMA). Percentage of cells positive for ASCL1 (A) and PHOX2B (P) for each cell line after sympatho-adrenal priming are as follows: H1: A, 61.2 ± 16.7; P, 57.1 ± 24.9; AP, 48.7 ± 20.9. H9: A, 35.1 ± 19.6; P, 67.7 ± 6.2; AP, 32.3 ± 16.7. Shef6: A, 23.4 ± 17.4; P, 26.2 ± 11.9; AP, 14.1 ± 9.9. Miff-1: A, 19.8 ± 21.2; P, 26.9 ± 9.1; AP, 10.5 ± 7.2. Yale6: A, 77.6 ± 13.2; P, 88.4 ± 8.9; AP, 73.2 ± 12.1. Scale bar represents 100 μm. See also Figures S1 and S2.

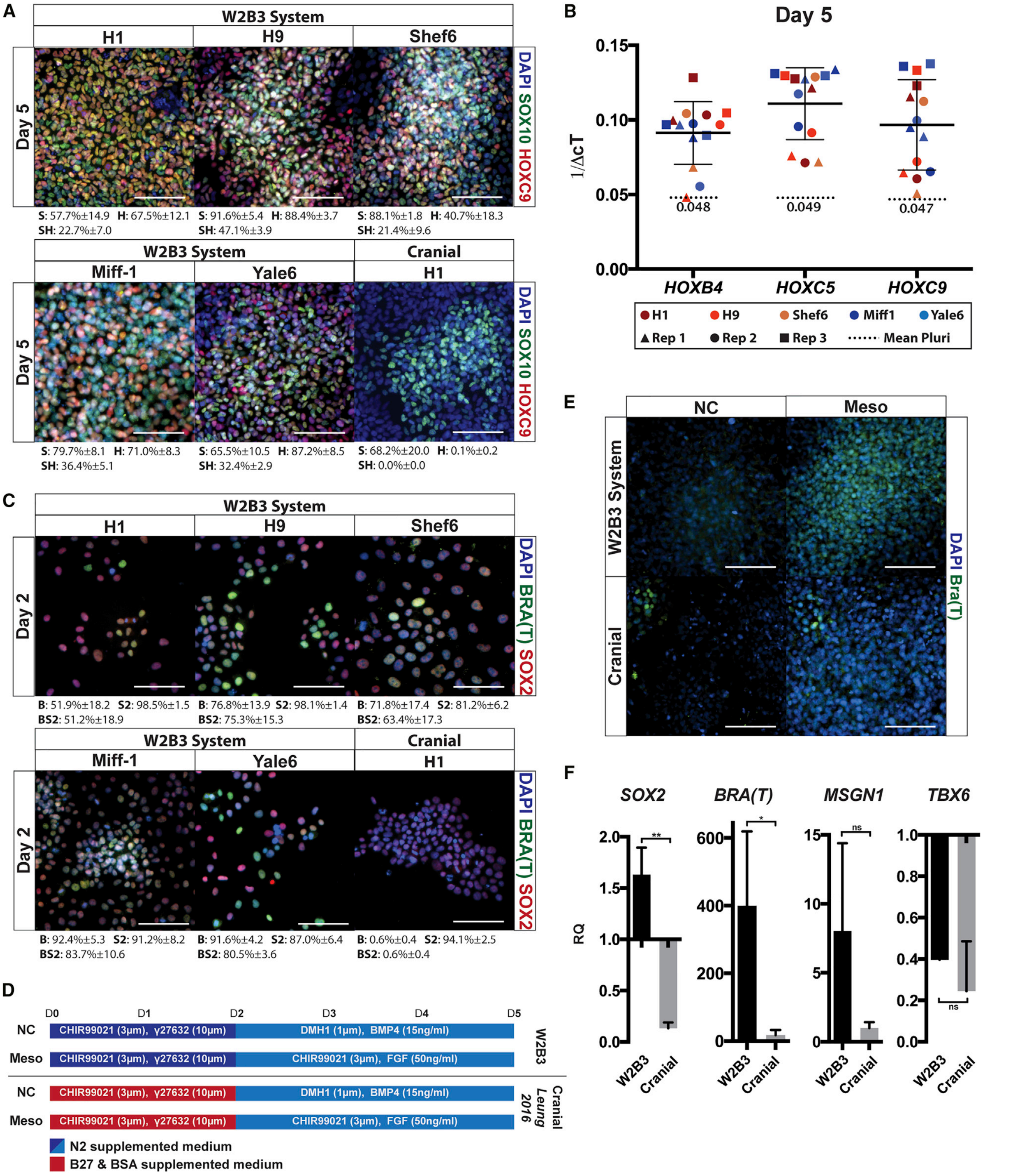

Figure 3. Characterization of Day 2 Neural Crest Progenitors

(A) Immunofluorescence microscopy showing co-expression of the caudal marker HOXC9 with SOX10 at day 5 in the W2B3 system across five hPSC lines but not after cranial neural crest induction using H1. Quantification calculated from one independent experiment per cell line. S, SOX10 positive, H, HOXC9 positive.

(legend continued on next page)

HOXA11, and *HOXD13* was observed. In all cases, PD17 treatment repressed *HOX* gene expression (Figure 4E).

Finally, because WNT signaling was found to modulate trunk versus cranial fate in Gomez et al. (2019b), we investigated the effect of different levels of WNT stimulation in the W2B3 system. It was found that only with a reduction in the concentration of the WNT mimic CHIR99021 could expression of the rostral marker *OTX2* be seen (Figure 4F). This suggests that within the first 2 days of neural crest induction, WNT signaling dictates whether a cranial or trunk fate is adopted, while FGF signaling modulates axial identity within the trunk compartment (Figure 4G).

DISCUSSION

Here, we present a system for the *in vitro* generation of trunk neural crest from hPSCs. The fully defined and xeno-free nature of the W2B3 protocol makes it ideal for studying the differentiation of hPSCs into trunk neural crest, as well as generating cells for use clinically. Strikingly, this system is highly robust across multiple hPSC lines, both ESCs and induced PSCs. This is in contrast to previously published protocols for neural crest induction, which appear to be cell-line specific and therefore difficult to implement across different cell lines. Despite this, there is still a small degree of variability in outcome across all cell lines (e.g., ±10% SOX10-positive cells in H1), which may be due to a variable GSK3β inhibition response caused by cell-cycle profiles of hPSCs prior to induction (Laco et al., 2018). This may also be the reason why leaving cultures until they are highly confluent prior to induction enhances efficiency. Expression of the neural marker *PAX6* remained very low after induction in all five cell lines (H1-ΔcT pluri 15.5; NC 13.0) suggesting very little contamination of cultures with neural cell types. Although *FOXD3* expression is only upregulated by approximately 10-fold in most cell lines, this is in large part due to fairly high levels of transcripts detected in pluripotent cell cultures (H1-ΔcT pluri 6.8; NC 4.7).

The robustness exhibited in induction of neural crest extends to the *in vitro* derivation of a wide range of terminal derivatives normally formed by neural crest *in vivo*, including melanocytes, peripheral neurons, and glia among others. For this project, we adapted the melanocyte differentiation protocol reported in Mica et al. (2013) so that a cell-sorting step is not required. Using this improved approach, it was possible to generate pigmented melanocytes with both the W2B3 trunk neural crest induction system and a previously published cranial neural crest protocol. During this process, it took longer for the cranial neural crest to achieve pigmentation, but whether this indicates a functional difference at the cellular level is unclear. Most notable was the generation of sympathoadrenal lineages from neural crest cells derived using the W2B3 system and the contrasting inability of cranial neural crest to do the same in these conditions, supporting an identity corresponding to trunk neural crest.

Interestingly, when examining the potential of W2B3-derived neural crest to form classic cranial ectomesenchymal derivatives, we were unable to generate cells of a chondrogenic fate, but smooth muscle and osteogenic fates were readily obtained. This result only partially supports the trunk character of these neural crest cells. However, the restricted potential of trunk neural crest to form ectomesenchymal derivatives after heterotopic grafts as shown by Le Douarin et al. (1977) and Nakamura and Ayer-le Lievre (1982) does not appear to be reflected *in vitro* where explanted chick trunk neural crest exposed to specific growth conditions can generate smooth muscle,

(B) qPCR data depicting *HOX* gene expression across five hPSC lines at day 5 of W2B3 neural crest induction. Each hPSC line is denoted by color, and each independent experiment is shown as a different shape (see key). Data are presented as 1/ΔcT as *HOX* genes are essentially not expressed in pluripotent cells, rendering fold change inappropriate.
(C) Immunofluorescence microscopy showing co-expression of neural marker SOX2 and mesodermal marker Brachyury (BRA) after 2 days of W2B3 neural crest induction. When using an established cranial neural crest induction protocol (Leung et al., 2016), Brachyury is not detected. Quantification calculated from two independent experiments per cell line. Quantification is presented below each image. B, Brachyury positive; S2, SOX2 positive; BS2, Brachyury/SOX2 positive.
(D) Schematic depicting *in vitro* mesoderm potential assay whereby cultures were switched into medium containing CHIR99021 (3 μM) and FGF2 (50 ng mL^{-1}) at day 2 of induction using the W2B3 system or day 2 of induction using an established cranial neural crest induction protocol (Leung et al., 2016).
(E) Immunofluorescence microscopy showing detection of the mesoderm marker Brachyury in day 5 cultures subjected to either the W2B3 system, a previously published cranial protocol, or the first 2 days of each protocol followed by sustained WNT and FGF exposure. Brachyury is broadly expressed after day 2 W2B3 neural crest precursors are subjected to mesodermal conditions but not in the case of cranial neural crest precursors.
(F) qPCR corresponding to the *in vitro* mesoderm potential assay; *Brachyury* is significantly more upregulated in the W2B3 system than in the cranial neural crest system but other mesoderm markers are not. Mean ΔcT values for each condition are as follows: *SOX2:* W2B3, 5.7; cranial, 8.5. *Bra(T)*: W2B3, 6.5; cranial, 11.4. *MSGN1*: W2B3, 11.9; cranial, 14.7. *TBX6*: W2B3, 11.4; cranial, 12.2.
Scale bars represent 100 μm. *$p < 0.05$, **$p < 0.01$. ns, not significant. See also Figure S3.

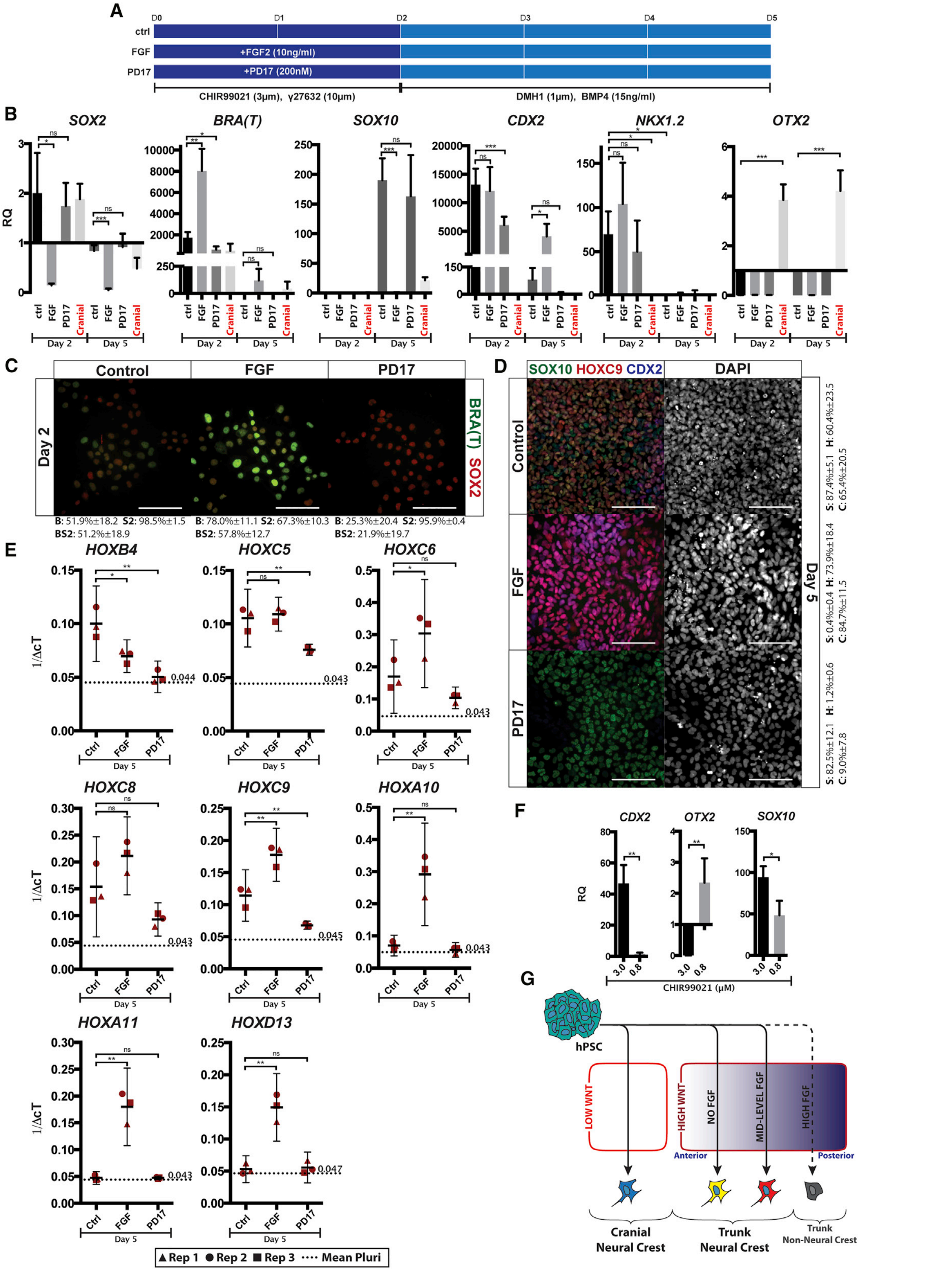

(legend on next page)

osteocytes, and even chondrocytes (Abzhanov et al., 2003; McGonnell and Graham, 2002). This highlights a need to identify the minimal signaling requirements for induction of specific derivatives, so that *in vitro* experiments can inform on the relevant differences in cellular potential at play during *in vivo* development. We anticipate that future work will define the minimal signaling (culture) conditions that differentially enable the proper terminal differentiation of cranial or trunk neural crest *in vitro*.

During embryonic development, caudal extension of the embryo is achieved by the proliferation and differentiation of an axial stem cell pool that exists at the border of the primitive streak. These cells express *SOX2* and *Brachyury* in a heterogeneous fashion and have the capacity to differentiate into either neural or mesodermal tissue in a manner that is largely dependent on their location within the primitive streak border region (Wymeersch et al., 2016). There is evidence to suggest that in mice and in avians, trunk neural crest may at least in part be derived from these axial progenitors (Catala et al., 1995; Rodrigo Albors et al., 2018; Tzouanacou et al., 2009; Wymeersch et al., 2016), and a recent publication shows that hPSC-derived axial progenitors are able to generate trunk neural crest (Frith et al., 2018). In the embryo, PAX7 expression labels the entirety of both neural folds. This includes the most posterior, and presumably newly formed, open neural plate border that surrounds the node, anterior primitive streak, and the $SOX2^{+}$/$Brachyury^{+}$ axial progenitor zone (HH stage 4+ to 6 chicken embryos; Basch et al., 2006; Otto et al., 2006; Vadasz et al., 2013). This suggests that neural crest derived from axial progenitors *in vivo* would either be from contributions to already formed, PAX7 positive, neural folds by moving laterally from the NSB or anterior CLE, or alternatively axial progenitors could be the source of cells that generate the neural folds at their most posterior end. It is unclear whether there are cells in the CLE that express *SOX2, Brachyury*, and *PAX7* simultaneously, but it is possible that the modest upregulation of *PAX7* transcripts seen at day 2 of induction using our system could be a reflection of this. Other possibilities are that the neural crest in this region of the embryo could be derived from outside the SOX2 and Brachyury expressing region. Evidence from various groups and model organisms has demonstrated the potential that the non-neural ectoderm has to contribute to neural crest (Mancilla and Mayor, 1996; Moury and Jacobson, 1990; Pieper et al., 2012; Selleck and Bronner-Fraser, 1995; Streit and Stern, 1999; Yardley and García-Castro, 2012), and SOX2 negative, caudal epiblast has not been ruled out as a potential source for neural crest. None of these sources are mutually exclusive from one another, and it is possible that all contribute to the formation of trunk neural crest.

Co-expression of *SOX2* and *Brachyury* at day 2 of induction using the W2B3 protocol, as well as the presence of *NKX2.1* transcripts (Rodrigo Albors et al., 2018), suggests that the trunk neural crest progenitors present *in vitro* at this stage may correspond in some way to the axial progenitors of the embryo. This is further supported by the absence of *Brachyury* expression at the corresponding stage in cranial neural crest induction. These trunk neural crest progenitors have the ability to respond to continued WNT and FGF signaling by retaining *Brachyury* expression to day 5 of induction, but *SOX2* is also expressed and other mesoderm markers are not. It is likely that in these conditions, the cells continue to exist in an early progenitor state rather than differentiating toward mesoderm. It is possible that different conditions might achieve mesoderm induction, or alternatively the day 2 progenitors may be functionally distinct from those reported by others that retain mesoderm potential, despite

Figure 4. Axial Identity Is Modulated by FGF and WNT Signaling

(A) Schematic depicting FGF modulation during the first 2 days of W2B3 neural crest induction.

(B) qPCR analysis of gene expression associated with neural (*SOX2*), mesodermal (*BRA*), neural crest (*SOX10*), caudal (*CDX2, NKX1.2*), and rostral (*OTX2*) cell identity. Samples were taken at day 2 and day 5 of neural crest induction after exposure to either FGF2 (10 ng mL^{-1}) or the FGF inhibitor PD17 (200 nM) for the first 48 h of induction. Expression at equivalent point during cranial neural crest induction is also shown.

(C) Immunofluorescence microscopy showing detection of SOX2 and BRA at day 2 of induction after exposure to FGF or PD17. Quantification calculated from three independent experiments. B, Brachyury positive; S2, SOX2 positive; BS2, Brachyury/SOX2 positive.

(D) Immunofluorescence microscopy demonstrating expression of SOX10, HOXC9, and CDX2 at day 5 of induction when treated with FGF or PD17 for the first 48 h. Quantification is calculated from three independent experiments and presented to the right of the panel. S, SOX10 positive; H, HOXC9 positive; C, CDX2 positive.

(E) qPCR analyses of *HOX* gene expression at day 5 of induction in response to treatment with FGF or PD17 during the first 48 h. Data are presented as 1/ΔcT.

(F) qPCR showing transcription of caudal gene *CDX2*, rostral gene *OTX2*, and neural crest marker *SOX10* at day 5 of induction with either 3.0 μM or 0.8 μM CHIR99021 during the first 48 h.

(G) Schematic demonstrating the manner in which WNT and FGF signaling direct cells toward different axial identities. Signals act in concert with one another rather than in series.

Scale bars represent 100 μm. $^{*}p < 0.05$, $^{**}p < 0.01$, $^{***}p < 0.001$. ns, not significant. Experiments carried out using H1.

co-expression of Brachyury and SOX2. Another possibility is that by day 2, they have lost the ability to differentiate into mesoderm but that they have this capacity at an earlier time point. Interestingly, manipulation of FGF signaling during the first 48 h of induction has a dramatic effect on the balance of expression between *Brachyury* and *SOX2*. This raises the possibility that *in vitro* FGF is the primary factor determining to which zone of the primitive streak border region these cells most closely correspond. It is also in keeping with evidence that high FGF promotes *Brachyury* expression in the tailbud (Olivera-Martínez et al., 2012). Finally, it should be stated that, although in this case it appears that we are generating trunk neural crest via an axial progenitor-like intermediate, this does not rule out the possibility that trunk neural crest may also be derived from hPSC via an alternative route.

The data presented here implicate FGF signaling as a major determinant in axial-specific gene expression within the trunk compartment. Cell-line-independent variation was observed in the expression of *HOX* genes at day 5 of trunk neural crest induction and, because of the reported role of FGF in inducing a caudal fate, the effect of FGF manipulation on *HOX* gene expression was analyzed. The inhibitory effect of the small-molecule FGFR1/3 inhibitor PD17 on caudal *HOX* gene expression and the promoting effect of FGF specifically on posterior *HOX* genes indicate that the variable *HOX* expression across independent experiments could be due to variation in endogenous FGF output within the culture.

These data also show that FGF manipulation can be used to derive trunk neural crest that corresponds to different axial identities, something that could be useful in the generation of specific terminal derivatives such as endocrine cells or sacral neural crest-derived enteric neurons. In the embryo, cells within the progenitor zone responsible for embryonic extension exhibit a temporally controlled activation of *HOX* genes in the 3′-5′ direction within each *HOX* cluster (Deschamps and Duboule, 2017; Limura and Pourquié, 2006; Neijts et al., 2016). This so-called "*HOX* clock" ensures that cells exiting the primitive streak early during extension exhibit an anterior *HOX* code, whereas those that are retained within the primitive streak for longer exhibit a progressively posterior *HOX* code. In both mouse and chicken embryos, *Fgf8* is highly expressed at the primitive streak and node but tapers off anteriorly from this position (Wilson et al., 2009). Manipulation of FGF signaling in the system described here may be mimicking this process and directing cells toward an identity that corresponds to different positions within this embryonic region, thus explaining the effect on both axial position markers (*HOX* genes, *CDX2*) and axial progenitor markers (*SOX2, Brachyury)*. If this is the case, then it may be that timed treatment with FGF could be used to elicit a similar response. While expression of the most posterior *HOX* genes was only achieved in the absence of SOX10 expression, this was after treatment with high levels of FGF2 (10 ng mL^{-1}). We predicted that titration of FGF2 during the first 48 h of induction might reveal a concentration where sacral *HOX* expression is induced in the presence of *SOX10*, however this was not the case (data not shown). Instead, it may be necessary to extend the period of time cells are exposed to CHIR and endogenous FGF signals (i.e., 3 days instead of 2 days), or alternatively treat cultures with GDF11 as has been done in a different system (Lippmann et al., 2015).

Although expression of markers associated with a caudal cell fate could be modulated through manipulation of FGF signaling, expression of the cranial marker *OTX2* did not significantly change in the presence of FGF or PD17. A switch in identity from trunk to cranial neural crest could only be achieved with a reduction in the concentration of the WNT mimic CHIR99021 from 3 μM to 0.8 μM. This suggests that FGF signaling is not involved in the decision between a cranial and trunk fate. It is also in keeping with studies in amphibians that show Wnt signaling represses *otx2* (*Otx-A*) expression and promotes expression of posterior neural genes (McGrew et al., 1995) as well as a recent study that describes how WNT directs cells toward a posterior identity in a manner that is time dependent and relies on *CDX2* (Metzis et al., 2018). It also explains why titration of CHIR99021 results in a much-discussed "double peak" of neural crest induction efficiency (Gomez et al., 2019b).

Thus, our data suggest that through careful control of these two factors early during neural crest induction (and with addition of retinoic acid if vagal identity is required as has been previously published; Fattahi et al., 2016; Mica et al., 2013; Frith et al., 2018), it is possible to generate neural crest corresponding to a wide range of axial positions within the embryo. The concentration of CHIR99021 required for cranial neural crest induction in this system (0.8 μM) is lower than that reported for cranial neural crest induction protocols that utilize BSA-containing medium (3.0 μM; Mica et al., 2013; Leung et al., 2016) but equivalent to published protocols that are BSA free (Hackland et al., 2017; Tchieu et al., 2017), suggesting that BSA suppresses the potency of this small molecule, or the sensitivity of cells to it. Although any major deviations from 3 μM CHIR99021 result in a loss of *SOX10* expression (data not shown; excepting the lower, cranial neural crest induction *SOX10* expression peak), it is possible that small changes in the WNT signaling environment may also have an effect on axial identity within the trunk compartment without loss of neural crest identity. It may also be the case that changes in the FGF environment feed back into expression of WNT ligands and vice versa. To investigate this further, it will be necessary to look at

expression of known WNT and FGF effector genes ideally with simultaneous control of the signaling environment in a manner equivalent to the TDi control of BMP utilized in this protocol and described in Hackland et al. (2017).

Here, we present an extensively robust system that can be easily adapted to generate a range of different axial subtypes of human neural crest *in vitro*. This W2B3 protocol allowed for the identification of distinct roles for both WNT and FGF signaling in hPSC-derived neural crest identity, which is not only an essential step in understanding the process by which neural crest induction occurs but also demonstrates the advantages conferred by a fully defined, BSA-free system.

EXPERIMENTAL PROCEDURES

Pluripotent Stem Cell Culture

All cell lines were cultured in mTESR (STEMCELL Technologies) on Matrigel. Passaging was carried out using EDTA (Versene, Thermo Fisher Scientific no. 15040066).

Neural Crest Induction

W2B3 neural crest induction was achieved by treating hPSC cultures (4–5 days post passage; >95% confluency) with Accutase (STEMCELL Technologies no. 07920) at 37°C until cells were visibly detaching (<5 min). Cells were triturated to a single-cell suspension (~10×) and seeded at 20,000 cells cm^{-2} onto Matrigel-coated dishes (Corning no. 354234) in DMEM F12 (Thermo Fisher Scientific no. 11320-033) with N2 (Thermo Fisher Scientific no. 17502048), γ-27632 (10 μM; Tocris no. 1254) and CHIR99021 (3 μM; Tocris no. 4423). After 2 days, cultures were switched into DMEM F12 with N2, DMH1 (1 μM; Tocris no. 4126) and BMP4 (15 ng mL^{-1}; R&D no. 314-BP). The culture medium was changed daily. Cranial neural crest induction was carried out as described in Gomez et al. (2019a), which is an adapted version of the protocol first described in Leung et al. (2016).

Terminal Differentiation of Neural Crest

Differentiation of neural crest into melanocytes was achieved using a simplified version of the protocol described in Mica et al. (2013) (Figure S1). After 5 days of neural crest induction, cultures were switched into a “melanocyte priming” medium consisting of DMEM F12, N2 supplement, BMP4 (15 ng mL^{-1}) and Endothelin 3 (100 nM) and cultured for a further 5 days. After this period, cells were detached using Accutase and seeded back onto poly-L-ornithine-treated plates coated with laminin and fibronectin in a “melanocyte maturation” medium consisting of 30% low-glucose DMEM, 20% MCDB 201, 50% NeuroBasal medium, 0.5× N2 supplement, 0.5× B27, linoleic acid (0.5 mg mL^{-1}), ascorbic acid (0.5 mM), cAMP (250 μM), TPA (25 nM), cholera toxin (10 pM), CHIR99021 (1.5 μM), SCF (25ng mL^{-1}), EDN3 (50 nM), FGF2 (2 ng mL^{-1}), and BMP4 (12.5 ng mL^{-1}). After 4–5 days, expression of MITF and SOX10 was observed. Cultures were passaged a further 2–3 times to observe large numbers of pigmented cells. Appearance of pigmented cells was dependent on both time and high confluency and as such, cultures may be left for up to 2 weeks without passage in order to achieve the required confluency (this was necessary in the case of cranial neural crest; Figures S1 and S2). Passaging was required when cell numbers were in excess of that needed for a dense monolayer or when budding of pigmented spheroids occurred.

Differentiation into peripheral neurons and glia was achieved by following the protocols described in Lee et al. (2010). Differentiation into sympatho-adrenal lineages was carried out using a method adapted from Oh et al. (2016). Cells were detached on day 5 using Accutase and seeded onto Matrigel-coated plates at a density of 200,000 cells cm^{-2} in SA priming medium consisting of DMEM F12, N2, B27, NEAA, Glutamax, BMP4 (50 ng mL^{-1}), purmorphamine (1.5 μM; Tocris no. 4551) and SHH (50 ng mL^{-1}; R&D no. 1845-SH) supplemented with γ-27632 (10 μM) for the first 24 h. This medium was changed every 48 h for a total of 5 days at which point PHOX2B and ASCL1 expression was observed. Expression of tyrosine hydroxylase and peripherin was then achieved by again detaching the cells using Accutase and seeding onto Matrigel-coated plates at a density of 100,000 cells cm^{-2} in SA maturation medium consisting of DMEM F12, N2, B27, non-essential amino acid solution, Glutamax, GDNF (10 ng mL^{-1}; Peprotech no. 450-10), BDNF (10 ng mL^{-1}; Peprotech no. 450-02), and NGF (10 ng mL^{-1}; Peprotech no. 450-01) supplemented with γ-27632 (10 μM) for the first 24 h and refreshed every 48 h for 7 days.

Differentiation into osteocytes, chondrocytes, and smooth muscle was achieved following the protocols described in Leung et al. (2016). In cases where chondrocytic differentiation was unsuccessful, no data are shown because cohesive pellets did not form.

Immunofluorescence Microscopy

Immunofluorescence microscopy was carried out by fixing cells in 4% paraformaldehyde for 8 min followed by permeabilization in 0.4% Triton X-100 for 10 min and blocking in 10% fetal calf serum in PBS with Tween 20 (0.05%). Primary antibodies (Table S1) were incubated overnight at 4°C in blocking buffer, and secondary antibodies were incubated for 1 h at room temperature. Imaging and image analysis were carried out using a Nikon Eclipse Ti and Nikon Elements software, respectively. Image analysis was carried out using the bright spot detection algorithm, and in each experiment a negative control without primary antibody was used to set the positive/negative threshold.

qPCR

Total RNA was extracted using Trizol reagent (Life Technologies no. 15596026), and cDNA was generated using a PrimeScript RT-PCR Kit (Clontech no. RR014B). qPCR was carried out using SYBR Premix Ex Taq II (Takara no. RR820L). All qPCR data represent three independent experiments, and ΔcT values were calculated using a GAPDH control. Error bars represent standard deviation, and statistical analysis was carried out by unpaired t test. Primer pairs are listed in Table S2.

SUPPLEMENTAL INFORMATION

Supplemental Information can be found online at https://doi.org/10.1016/j.stemcr.2019.04.015.

AUTHOR CONTRIBUTIONS

M.I.G.-C. directed the study and edited the manuscript. M.I.G.-C. and J.O.S.H. conceived the project. J.O.S.H. developed the differentiation protocol, performed the experiments, and wrote the manuscript. N.S., R.M.C., and M.S.P. aided in terminal differentiation experiments. P.B.S., N.S., M.S.P., T.J.R.F., and G.A.G. aided in experiments investigating the characteristics of day 2 neural crest progenitors. P.B.S. aided in image analysis. T.J.R.F. adapted and developed the sympatho-adrenal terminal differentiation protocol. J.O.S.H. and M.S.P. adapted and developed the melanocyte differentiation protocol.

ACKNOWLEDGMENTS

We would like to thank Anestis Tsakiridis and Peter Andrews for their advice and for critically reading the manuscript. This work is supported by funding from the National Institutes of Health (NIH R01DE017914).

Received: January 8, 2019
Revised: April 15, 2019
Accepted: April 16, 2019
Published: May 14, 2019

REFERENCES

Abu-Bonsrah, K.D., Zhang, D., Bjorksten, A.R., Dottori, M., and Newgreen, D.F. (2018). Generation of adrenal chromaffin-like cells from human pluripotent stem cells. Stem Cell Reports *10*, 134–150.

Abzhanov, A., Tzahor, E., Lassar, A.B., and Tabin, C.J. (2003). Dissimilar regulation of cell differentiation in mesencephalic (cranial) and sacral (trunk) neural crest cells in vitro. Development *130*, 4567–4579.

Basch, M.L., Bronner-Fraser, M., and García-Castro, M.I. (2006). Specification of the neural crest occurs during gastrulation and requires Pax7. Nature *441*, 218–222.

Bolande, R. (1974). The neurocristopathies: a unifying concept of disease arising in neural crest maldevelopment. Hum. Pathol. *5*, 409–429.

Cambray, N., and Wilson, V. (2002). Axial progenitors with extensive potency are localised to the mouse chordoneural hinge. Development *129*, 4855–4866.

Catala, M., Teillet, M.A., and Le Douarin, N.M. (1995). Organization and development of the tail bud analyzed with the quail-chick chimaera system. Mech. Dev. *51*, 51–65.

Chibon, P. (1967). [Nuclear labelling by tritiated thymidine of neural crest derivatives in the amphibian Urodele *Pleurodeles waltlii* Michah]. J. Embryol. Exp. Morphol. *18*, 343–358.

Da Cruz, L., Fynes, K., Georgiadis, O., Kerby, J., Luo, Y.H., Ahmado, A., Vernon, A., Daniels, J.T., Nommiste, B., Hasan, S.M., et al. (2018). Phase 1 clinical study of an embryonic stem cell–derived retinal pigment epithelium patch in age-related macular degeneration. Nat. Biotechnol. *36*, 328–337.

Denham, M., Hasegawa, K., Menheniott, T., Rollo, B., Zhang, D., Hough, S., Alshawaf, A., Febbraro, F., Ighaniyan, S., Leung, J., et al. (2015). Multipotent caudal neural progenitors derived from human pluripotent stem cells that give rise to lineages of the central and peripheral nervous system. Stem Cells *33*, 1759–1770.

Deschamps, J., and Duboule, D. (2017). Embryonic timing, axial stem cells, chromatin dynamics, and the Hox clock. Genes Dev. *31*, 1406–1416.

Fattahi, F., Steinbeck, J.A., Kriks, S., Tchieu, J., Zimmer, B., Kishinevsky, S., Zeltner, N., Mica, Y., El-Nachef, W., Zhao, H., et al. (2016). Deriving human ENS lineages for cell therapy and drug discovery in Hirschsprung disease. Nature *531*, 105–109.

Frith, T.J.R., Granata, I., Wind, M., Stout, E., Thompson, O., Neumann, K., Stavish, D., Heath, P.R., Ortmann, D., Hackland, J.O.S., et al. (2018). Human axial progenitors generate trunk neural crest cells in vitro. Elife *7*, 134.

Funa, N.S., Schachter, K.A., Lerdrup, M., Ekberg, J., Hess, K., Dietrich, N., Honoré, C., Hansen, K., and Semb, H. (2015). β-Catenin regulates primitive streak induction through collaborative interactions with SMAD2/SMAD3 and OCT4. Cell Stem Cell *16*, 639–652.

Gomez, G.A., Prasad, M.S., Sandhu, N., Shelar, P.B., Leung, A.W., and García-Castro, M.I. (2019a). Human neural crest induction by temporal modulation of WNT activation. Dev. Biol. *449*, 99–106.

Gomez, G.A., Prasad, M.S., Wong, M., Charney, R.M., Shelar, P., Sandhu, N., Hackland, J., Hernandez, J.C., Leung, A.W., and García-Castro, M.I. (2019b). WNT/β-CATENIN modulates the axial identity of ES derived human neural crest. bioRxiv https://doi.org/10.1101/514570.

Gont, L.K., Steinbeisser, H., Blumberg, B., and De Robertis, E.M. (1993). Tail formation as a continuation of gastrulation: the multiple cell populations of the Xenopus tailbud derive from the late blastopore lip. Development *119*, 991–1004.

Gouti, M., Tsakiridis, A., Wymeersch, F.J., Huang, Y., Kleinjung, J., Wilson, V., and Briscoe, J. (2014). In vitro generation of neuromesodermal progenitors reveals distinct roles for Wnt signalling in the specification of spinal cord and paraxial mesoderm identity. PLoS Biol. *12*, e1001937.

Hackland, J.O.S., Frith, T.J.R., Thompson, O., Marin Navarro, A., García-Castro, M.I., Unger, C., and Andrews, P.W. (2017). Top-down inhibition of BMP signaling enables robust induction of hPSCs into neural crest in fully defined, xeno-free conditions. Stem Cell Reports *9*, 1043–1052.

His, W. (1868). Untersuchungen über die erste Anlage des Wirbelthierleibes (F.C.W. Vogel), pp. 1–293.

Kirino, K., Nakahata, T., Taguchi, T., and Saito, M.K. (2018). Efficient derivation of sympathetic neurons from human pluripotent stem cells with a defined condition. Sci. Rep. *8*, 12865.

Laco, F., Woo, T.L., Zhong, Q., Szmyd, R., Ting, S., Khan, F.J., Chai, C.L.L., Reuveny, S., Chen, A., and Oh, S. (2018). Unraveling the inconsistencies of cardiac differentiation efficiency induced by the GSK3β inhibitor CHIR99021 in human pluripotent stem cells. Stem Cell Reports *10*, 1851–1866.

Le Douarin, N.M., and Teillet, M.-A.M. (1974). Experimental analysis of the migration and differentiation of neuroblasts of the autonomic nervous system and of neurectodermal

mesenchymal derivatives, using a biological cell marking technique. Dev. Biol. *41*, 162–184.

Le Douarin, N.M., Teillet, M.A., and Le Lievre, C. (1977). Influence of the tissue environment on the differentiation of neural crest cells. Soc. Gen. Physiol. Ser. *32*, 11–27.

Le Douarin, N.M., Creuzet, S., Couly, G., and Dupin, E. (2004). Neural crest cell plasticity and its limits. Development *131*, 4637–4650.

Le Lievre, C.S., and Le Douarin, N.M. (1975). Mesenchymal derivatives of the neural crest: analysis of chimaeric quail and chick embryos. J. Embryol. Exp. Morphol. *34*, 125–154.

Lee, G., Chambers, S.M., Tomishima, M.J., and Studer, L. (2010). Derivation of neural crest cells from human pluripotent stem cells. Nat. Protoc. *5*, 688–701.

Leung, A.W., Murdoch, B., Salem, A.F., Prasad, M.S., Gomez, G.A., and García-Castro, M.I. (2016). WNT/beta-catenin signaling mediates human neural crest induction via a pre-neural border intermediate. Development *143*, 398–410.

Limura, T., and Pourquié, O. (2006). Collinear activation of Hoxb genes during gastrulation is linked to mesoderm cell ingression. Nature *442*, 568–571.

Lippmann, E.S., Williams, C.E., Ruhl, D.A., Estevez-Silva, M.C., Chapman, E.R., Coon, J.J., and Ashton, R.S. (2015). Deterministic HOX patterning in human pluripotent stem cell-derived neuroectoderm. Stem Cell Reports *4*, 632–644.

Lwigale, P.Y., Conrad, G.W., and Bronner-Fraser, M. (2004). Graded potential of neural crest to form cornea, sensory neurons and cartilage along the rostrocaudal axis. Development *131*, 1979–1991.

Mancilla, A., and Mayor, R. (1996). Neural crest formation in *Xenopus laevis*: mechanisms of Xslug induction. Dev. Biol. *177*, 580–589.

Marshall, A.M. (1878). The development of the cranial nerves in the chick. J. Cell Sci. *s2-18*, 10–40.

McGonnell, I.M., and Graham, A. (2002). Trunk neural crest has skeletogenic potential. Curr. Biol. *12*, 767–771.

McGrew, L.L., Lai, C.J., and Moon, R.T. (1995). Specification of the anteroposterior neural axis through synergistic interaction of the Wnt signaling cascade with noggin and follistatin. Dev. Biol. *172*, 337–342.

Menendez, L., Yatskievych, T.A., Antin, P.B., and Dalton, S. (2011). Wnt signaling and a Smad pathway blockade direct the differentiation of human pluripotent stem cells to multipotent neural crest cells. Proc. Natl. Acad. Sci. U S A *108*, 19240–19245.

Metzis, V., Steinhauser, S., Pakanavicius, E., Gouti, M., Stamataki, D., Ivanovitch, K., Watson, T., Rayon, T., Mousavy Gharavy, S.N., Lovell-Badge, R., et al. (2018). Nervous system regionalization entails axial allocation before neural differentiation. Cell *175*, 1105–1118.e17.

Mica, Y., Lee, G., Chambers, S.M., Tomishima, M.J., and Studer, L. (2013). Modeling neural crest induction, melanocyte specification, and disease-related pigmentation defects in hESCs and patient-specific iPSCs. Cell Rep. *3*, 1140–1152.

Miller, J.D., Ganat, Y.M., Kishinevsky, S., Bowman, R.L., Liu, B., Tu, E.Y., Mandal, P.K., Vera, E., Shim, J.-W., Kriks, S., et al. (2013). Human iPSC-based modeling of late-onset disease via progerin-induced aging. Cell Stem Cell *13*, 691–705.

Moury, J.D., and Jacobson, A.G. (1990). The origins of neural crest cells in the axolotl. Dev. Biol. *141*, 243–253.

Nakamura, H., and Ayer-le Lievre, C.S. (1982). Mesectodermal capabilities of the trunk neural crest of birds. J. Embryol. Exp. Morphol. *70*, 1–18.

Neijts, R., Amin, S., Van Rooijen, C., Tan, S., Creyghton, M.P., De Laat, W., and Deschamps, J. (2016). Polarized regulatory landscape and Wnt responsiveness underlie Hox activation in embryos. Genes Dev. *30*, 1937–1942.

Oh, Y., Cho, G.-S., Li, Z., Hong, I., Zhu, R., Kim, M.-J., Kim, Y.J., Tampakakis, E., Tung, L., Huganir, R., et al. (2016). Functional coupling with cardiac muscle promotes maturation of hPSC-derived sympathetic neurons. Cell Stem Cell *19*, 95–106.

Okuno, H., Renault Mihara, F., Ohta, S., Fukuda, K., Kurosawa, K., Akamatsu, W., Sanosaka, T., Kohyama, J., Hayashi, K., Nakajima, K., et al. (2017). CHARGE syndrome modeling using patient-iPSCs reveals defective migration of neural crest cells harboring CHD7 mutations. Elife *6*. https://doi.org/10.7554/eLife.21114.

Olivera-Martínez, I., Harada, H., Halley, P.A., and Storey, K.G. (2012). Loss of FGF-dependent mesoderm identity and rise of endogenous retinoid signalling determine cessation of body axis elongation. PLoS Biol. *10*, e1001415.

Otto, A., Schmidt, C., and Patel, K. (2006). Pax3 and Pax7 expression and regulation in the avian embryo. Anat. Embryol. *211*, 293–310.

Parker, H.J., Pushel, I., and Krumlauf, R. (2018). Coupling the roles of Hox genes to regulatory networks patterning cranial neural crest. Dev. Biol. *444* (*Suppl 1*), S67–S78.

Pieper, M., Ahrens, K., Rink, E., Peter, A., and Schlosser, G. (2012). Differential distribution of competence for panplacodal and neural crest induction to non-neural and neural ectoderm. Development *139*, 1175–1187.

Pomp, O., Brokhman, I., Ben-Dor, I., Reubinoff, B., and Goldstein, R.S. (2005). Generation of peripheral sensory and sympathetic neurons and neural crest cells from human embryonic stem cells. Stem Cells *23*, 923–930.

Rodrigo Albors, A., Halley, P.A., and Storey, K.G. (2018). Lineage tracing of axial progenitors using Nkx1–2CreER(T2) mice defines their trunk and tail contributions. Development *145*, dev164319.

Rothstein, M., Bhattacharya, D., and Simões-Costa, M. (2018). The molecular basis of neural crest axial identity. Dev. Biol. *444* (*Suppl 1*), S170–S180.

Selleck, M.A., and Bronner-Fraser, M. (1995). Origins of the avian neural crest: the role of neural plate-epidermal interactions. Development *121*, 525–538.

Simões-Costa, M., and Bronner, M.E. (2015). Establishing neural crest identity: a gene regulatory recipe. Development *142*, 242–257.

Streit, A., and Stern, C.D. (1999). Establishment and maintenance of the border of the neural plate in the chick: involvement of FGF and BMP activity. Mech. Dev. *82*, 51–66.

Tchieu, J., Zimmer, B., Fattahi, F., Amin, S., Zeltner, N., Chen, S., and Studer, L. (2017). A modular platform for differentiation of human PSCs into all major ectodermal lineages. Cell Stem Cell *21*, 399–410.e7.

Tsakiridis, A., and Wilson, V. (2015). Assessing the bipotency of in vitro-derived neuromesodermal progenitors. F1000Res. *4*, 100.

Tsakiridis, A., Huang, Y., Blin, G., Skylaki, S., Wymeersch, F., Osorno, R., Economou, C., Karagianni, E., Zhao, S., Lowell, S., and Wilson, V. (2014). Distinct Wnt-driven primitive streak-like populations reflect in vivo lineage precursors. Development *141*, 1209–1221.

Turner, D.A., Hayward, P.C., Baillie-Johnson, P., Rue, P., Broome, R., Faunes, F., and Martinez Arias, A. (2014). Wnt/beta-catenin and FGF signalling direct the specification and maintenance of a neuromesodermal axial progenitor in ensembles of mouse embryonic stem cells. Development *141*, 4243–4253.

Tzouanacou, E., Wegener, A., Wymeersch, F.J., Wilson, V., and Nicolas, J.-F. (2009). Redefining the progression of lineage segregations during mammalian embryogenesis by clonal analysis. Dev. Cell *17*, 365–376.

Vadasz, S., Marquez, J., Tulloch, M., Shylo, N.A., and García-Castro, M.I. (2013). Pax7 is regulated by cMyb during early neural crest development through a novel enhancer. Development *140*, 3691–3702.

Wilson, V., Olivera-Martínez, I., and Storey, K.G. (2009). Stem cells, signals and vertebrate body axis extension. Development *136*, 1591–1604.

Wymeersch, F.J., Huang, Y., Blin, G., Cambray, N., Wilkie, R., Wong, F.C., and Wilson, V. (2016). Position-dependent plasticity of distinct progenitor types in the primitive streak. Elife *5*. https://doi.org/10.7554/eLife.10042.

Yardley, N., and García-Castro, M.I. (2012). FGF signaling transforms non-neural ectoderm into neural crest. Dev. Biol. *372*, 166–177.

Stem Cell Reports, Volume *12*

Supplemental Information

FGF Modulates the Axial Identity of Trunk hPSC-Derived Neural Crest but Not the Cranial-Trunk Decision

James O.S. Hackland, Patrick B. Shelar, Nabjot Sandhu, Maneeshi S. Prasad, Rebekah M. Charney, Gustavo A. Gomez, Thomas J.R. Frith, and Martín I. García-Castro

Table S1. List of primary antibodies

Target	Source/Cat#	Dilution
SOX10	Santa Cruz/271163	1:200
PAX7	DSHB	1:20
Peripherin	Sigma-Millipore/ab1530	1:200
Peripherin	Santa Cruz/377093	1:200
ISL1	DSHB/39-4D5	1:100
Β-Tubulin	Santa Cruz/365791	1:300
BRN3a	Santa Cruz/8429	1:100
ASCL1	Santa Cruz/374104	1:1000
PHOX2B	Santa Cruz/376997	1:500
Tyrosine Hydroxylase (TH)	Santa Cruz/25269	1:500
S100B	Santa Cruz/393919	1:50
Vimentin	Sigma-Millipore/MAB3400	1:100
SMA	Sigma/A2547	1:100
HOXC9	Santa Cruz/81100	1:500
Bra(T)	R&D/AF2085	1:200
CDX2	DSHB/PCRP-1A3	1:250
MITF	R&D/AF5769	1:50

Table S2. List of primers

Transcript	Forward	Reverse
GAPDH	ATGGGTGTGAACCATGAGAA	GTTGTCATGGATGACCTTGG
SOX10	GAGGCTGCTGAACGAAAGTGA	GCGGCCTTCCCGTTCT
TFAP2A	GAGAGTAGCTCCACTTGGGTG	GTCGTGACGGTCCTCGC
SNAI2	CAGACCCTGGTTGCTTCAAG	GAGCCCTCAGATTTGACCTG
FOXD3	TCATCACCATGGCCATCCT	GGAAGCGGTTGCTGATGAAC
PAX3	GAACCCGGGCATGTTCAG	ACGGCACGGTGTTTCGA
PAX7	GCGACTCCGGATGTAGAGAA	ATCCTTCAGCAGCCTGTCC
PAX6	ACCCGGCAGAAGATTGTAGA	ACTCACATCCGTTGGACA
OCT4	TGCAGCTTAGCTTCAAGAACATG	TCAGCTTCCTCCACCCACTT
BRA(T)	AGGCTCCCGTCTCCTTCAGCAA	TGGCTGGTGATCATGCGCTGT
SOX2	TGCGAGCGCTGCACAT	GCAGCGTGTACTTATCCTTCTTCA
CDX2	GGAACCTGTGCGAGTGGAT	TGAAACTCCTTCTCCAGCTCC
NKX1.2	AAAGTTTGGCGGAGGTCGAA	GCAGCATCAGGGGTCTCC
OTX2	GAGAGGAGGTGGCACTGAAAA	GTTGTTGGCGGCACTTAGC
HOXB4	TACCCCTGGATGCGCAAAGTTC	TGGTGTTGGGCAACTTGTGG

HOXC5	ACAGATTTACCCGTGGATGAC	AGTGAGGTAGCGGTTAAAGTG
HOXC6	ATGAATTCGCACAGTGGGGT	TCCTTCTCCAGTTCCAGGGT
HOXC8	GATGAGACCCCACGCTCCG	TTCTAGTTCCAAGGTCTGATACCG
HOXC9	AGCACAAAGAGGAGAAGGC	CGTCTGGTACTTGGTGTAGG
HOXA10	GGATTCCCTGGGCAATTCCAAA	CAGTGTCTGGTGCTTCGTGT
HOXA11	GGCGGCTCCAGTGGC	CGCTGAAGAAGAACTCCCGT
HOXD13	ACGCTGGCTAACGGGTG	TTAGAGCCACATCCCCTGGA

Figure S1

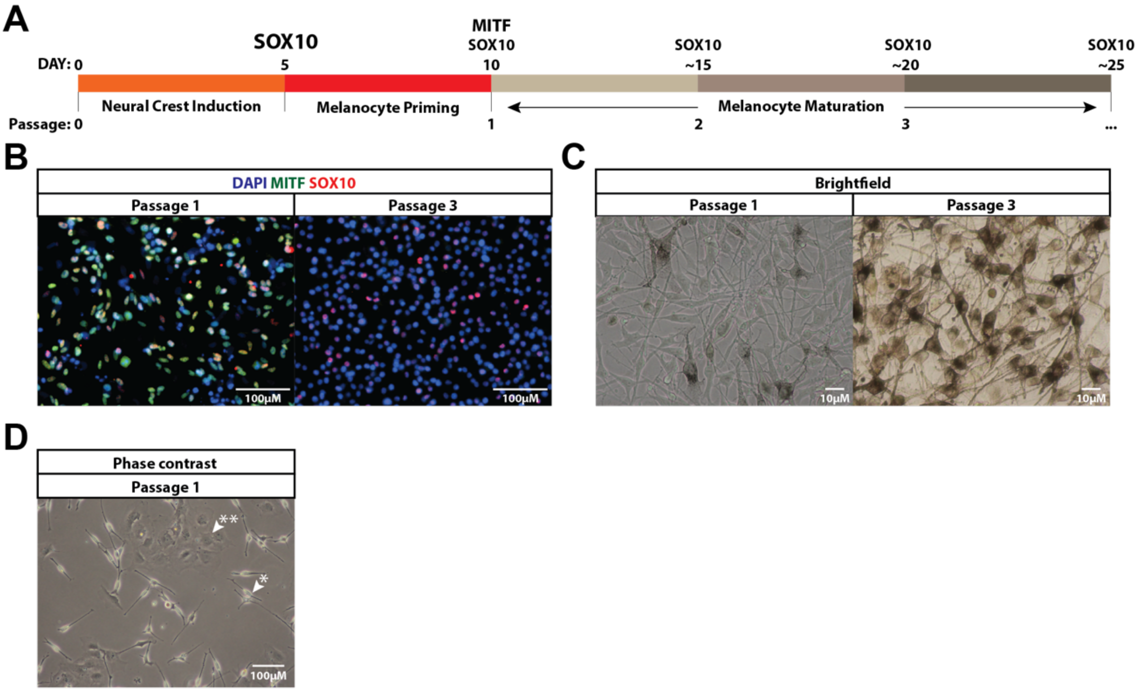

Figure S1. Melanocyte induction.

Related to Figure 2. (A) Schematic demonstrating melanocyte induction protocol. After 5 days of neural crest induction culture conditions were exchanged for a priming medium containing BMP4 and EDN3. After a further 5 days cells were detached and seeded into melanocyte maturation conditions (see Methods section). Subsequent passaging was carried out when cultures became highly confluent, approximately every 5 days. (B, C) After primed cells were detached and seeded into maturation conditions expression of MITF and SOX10 could be observed and a minority of cells exhibited pigmentation. Continued culture in these conditions resulted in a loss of MITF expression and a dramatic increase in the proportion of pigmented cells. Images of pigmented cells (C) were taken using a 20x objective on a Nikon TS100 inverted microscope with the condenser set to brightfield (i.e. no phase ring). (D) Low magnification phase contrast imaging (i.e. using a phase ring) at passage 1 reveals glowing, motile melanocyte progenitors (*) and low flat cells not of the melanocyte lineage (**). The latter terminally differentiate and are eventually outgrown and lost with passaging. Images shown are from melanocyte induction using Miff-1-derived neural crest (B, C) and H1-derived neural crest (D).

Figure S2

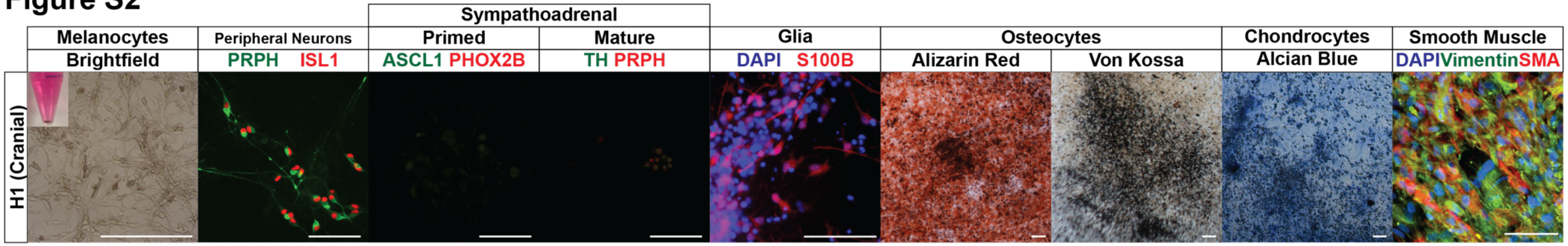

Figure S2. Terminal differentiation of cranial neural crest

Related to Figure 2. Terminal differentiation of cranial neural crest generated using a previously published protocol (Leung et al., 2016).

Figure S3

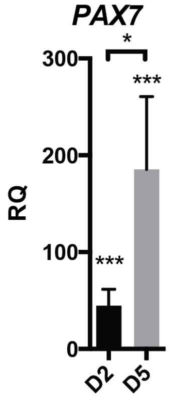

Figure S3. ***PAX7*** **expression in day 2 neural crest progenitors**

Related to Figure 3. qPCR data depicting expression of *PAX7* at day 2 and day 5 of neural crest induction using the W2B3 protocol in H1.
